# Nicotine Enhances Goal-Tracking in Ethanol and Food Pavlovian Conditioned Approach Paradigms

**DOI:** 10.3389/fnins.2021.561766

**Published:** 2021-08-18

**Authors:** Hailley Angelyn, Gregory C. Loney, Paul J. Meyer

**Affiliations:** Behavioral Neuroscience Program, Department of Psychology, State University of New York at Buffalo, Buffalo, NY, United States

**Keywords:** nicotine, alcohol, conditioned approach, goal-tracking, cue-reactivity

## Abstract

**Rationale:**

Nicotine promotes alcohol intake through pharmacological and behavioral interactions. As an example of the latter, nicotine can facilitate approach toward food- and alcohol-associated stimuli (“sign-tracking”) in lever-Pavlovian conditioned approach (PavCA) paradigms. However, we recently reported that nicotine can also enhance approach toward locations of reward delivery (“goal-tracking”) triggered by ethanol-predictive stimuli when the location of ethanol delivery is non-static (i.e., a retractable sipper bottle).

**Objective:**

To determine whether the non-static nature of the reward location could have biased the development of goal-tracking in our previous study (Loney et al., 2019); we assessed the effect of nicotine in a lever-PavCA paradigm wherein the location of ethanol delivery was static (i.e., a stationary liquid receptacle). Then, to determine whether nicotine’s enhancement of goal-tracking is unique to ethanol-predictive stimuli, we assessed the effect of systemic nicotine on approach triggered by food-predictive stimuli in a lever-PavCA paradigm.

**Methods:**

Long–Evans rats were used in two PavCA experiments wherein a lever predicted the receipt of ethanol (15% vol/vol; experiment 1) or food (experiment 2) into a stationary receptacle. Prior to testing, rats were administered nicotine (0.4 mg/kg subcutaneously) or saline systemically.

**Results:**

In both experiments, nicotine increased measures of goal-tracking, but not sign-tracking.

**Conclusion:**

Nicotine can facilitate approach to reward locations without facilitating approach to reward-predictive stimuli. As such, conceptualization of the mechanisms by which nicotine affects behavior must be expanded to explain an enhancement of goal-tracking by nicotine.

## Introduction

While there is limited evidence that the primary reinforcing properties of nicotine alone serve to drive drug-taking (Dar and Frenk, 2004; Caggiula et al., 2009), there is substantial evidence that both smoking and smoking concomitant behaviors are principally controlled by nicotine’s interactions with reward-associated stimuli (for review see Caggiula et al., 2009). When presented to smokers, nicotine-associated stimuli can maintain smoking behavior independently of nicotine intake (Donny et al., 2007), induce craving for nicotine (Carter and Tiffany, 1999), evoke conditioned physiological responses (Carter and Tiffany, 1999; Erblich et al., 2011; Winkler et al., 2011), and are necessary to yield reports of the rewarding (Rose et al., 2000) or euphoric (Dar et al., 2007) effects of nicotine. Similarly, nicotine-associated stimuli in rodent paradigms are necessary to maintain self-administration of nicotine (Caggiula et al., 2009), increase the motivation to obtain nicotine (Chaudhri et al., 2007), slow the extinction of operant responding for nicotine, and reinstate operant self-administration of nicotine after extinction (LeSage et al., 2004; Feltenstein et al., 2012; Versaggi et al., 2016). Notably, nicotine’s impact on operant responding is not limited solely to nicotine-associated stimuli, as nicotine produces similar effects on responding in the presence of stimuli that are associated with non-nicotine rewards (Caggiula et al., 2009), including alcohol (Le et al., 2003), which is commonly coabused with nicotine (Falk et al., 2006; Weinberger et al., 2016, 2015).

The basis for such interactions between nicotine and reward-associated stimuli may be due to nicotine’s incentive-amplifying properties that act on reward-associated stimuli (Caggiula et al., 2009; Palmatier et al., 2014). That is, by enhancing the motivational (i.e., incentive) value of reward-associated stimuli, nicotine increases the ability of such stimuli to trigger reward-seeking. These properties not only influence maintenance and relapse to smoking, but may also be responsible for the co-occurence of smoking and drinking, which clinical studies indicate are associated with a higher risk of relapse to alcohol abuse (Weinberger et al., 2015). However, it is difficult to distinguish whether responding is amplified as a result of nicotine affecting the incentive value of the reward-associated stimulus or the reinforcing value of the reward itself. To acheive this distinction, an effect of nicotine must be demonstrated using measures that reflect the incentive value of a stimulus.

One such measure of the incentive value of a stimulus is the extent to which it is able to elicit approach, bias attention, and spur motivated responses (Robinson et al., 2014). This property can be captured by Pavlovian conditioned approach (PavCA) paradigms in which approach is assessed in response to presentations of a stimulus that immediately precedes (i.e., predicts) the response-independent receipt of a reward. Because delivery of the reward is not contingent on the animal’s behavior, approach is directly under the control of the predictive stimulus. When the stimulus is not localizable, e.g., a tone, conditioned approach is singularly directed toward the reward location; we refer to this as a “simple”-PavCA paradigm. During simple-PavCA, attribution of incentive value to the stimulus is indicated by the magnitude of approach and interaction with the reward location (“goal-tracking”) elicited by the stimulus (Holland, 1977; Meyer et al., 2014). Conversely, when the stimulus is localizable, e.g., a lever, conditioned approach can be directed toward either the goal or the stimulus; we refer to this as a “lever”-PavCA paradigm (Flagel et al., 2007; Meyer et al., 2012). Within a lever-PavCA paradigm, attribution of incentive value to the stimulus is indicated by the degree of approach and interaction with the stimulus itself (“sign-tracking”), as opposed to the reward location. Notably, there is debate whether goal-tracking in a lever-PavCA paradigm represents the learned predictive value of the stimulus (Flagel et al., 2011; Robinson et al., 2014) and thus represents cognitive, goal-directed behavior (Pitchers et al., 2017a, 2018; Sarter and Phillips, 2018) or whether it represents attribution of incentive value to the reward location instead of the reward-associated stimulus, such that either the stimulus or location can serve as an individual’s “prepotent cue” (Mahler and Berridge, 2009; DiFeliceantonio and Berridge, 2012, 2016; Palmatier et al., 2014). Nonetheless, support for the traditional hypothesis that nicotine amplifies the incentive value of reward-associated stimuli would be demonstrated by an effect of nicotine on goal-tracking during simple PavCA paradigms and on sign-tracking during lever-PavCA paradigms.

Several studies are consistent with these expectations. For example, Maddux and Chaudhri (2017) found that administration of nicotine prior to testing in a simple PavCA paradigm increases the number of goal-tracking responses evoked by an ethanol-associated compound light/tone stimulus that is non-localizable in Long–Evans rats. Further, we have previously demonstrated that administration of nicotine prior to testing in a lever-PavCA paradigm increases sign-tracking to a lever that predicts a banana-flavored food pellet in Sprague–Dawley rats (Versaggi et al., 2016), and similarly, others have found that nicotine increases sign-tracking to a lever that predicts liquid sucrose (Palmatier et al., 2014; Stringfield et al., 2017) or water (Guy and Fletcher, 2014), as well as a lit nosepoke receptacle that predicts liquid sucrose (Palmatier et al., 2013), all in Sprague–Dawley rats. Additionally, self-administration of nicotine enhances sign-tracking, but not goal-tracking, in a 4-CS PavCA paradigm in male Sprague–Dawley rats wherein separate lever and tone stimuli both predict food reward in an attempt to eliminate the competing nature of sign-tracking and goal-tracking (Overby et al., 2018).

Notably, however, the results of two recent studies have reported findings that are inconsistent with the anticipated effects of nicotine in lever-PavCA paradigms. Specifically, Stringfield et al. (2017) found that nicotine increased both goal-tracking and sign-tracking responses in male Sprague–Dawley rats evoked by a lever that predicted liquid sucrose. Further, we found that nicotine selectively enhanced goal-tracking and not sign-tracking in response to a lever-CS+ that predicted access to a retractable sipper bottle containing ethanol, without affecting the response to a non-predictive lever-CS, in male Long–Evans rats (Loney et al., 2019). By demonstrating that nicotine can affect goal-tracking, these findings suggest that the traditional hypothesis of nicotine’s incentive-amplifying effects on reward-associated stimuli is an incomplete account of the mechanisms by which nicotine affects behavior. Furthermore, because the propensity to sign-tracking or goal-tracking in rodents has been associated with differences in liability for relapse (Robinson et al., 2014; Pitchers et al., 2017b), as well as functional differences in dopaminergic and cholinergic neuromodulatory systems (Pitchers et al., 2017a), it is possible that nicotine acts through disparate neuromechanisms to affect sign-tracking and goal-tracking. Understanding the differences in these mechanisms could impact the treatment of human nicotine disorders, as there is accumulating evidence for similar phenotypic variability in conditioned approach and cholinergic capacity in humans (Garofalo and di Pellegrino, 2015; Sarter et al., 2016b; Joyner et al., 2018; Colaizzi et al., 2020).

However, there exists a significant amount of literature demonstrating that the development of conditioned approach in PavCA paradigms can be influenced by various experimental parameters (Palmatier et al., 2013; Guy and Fletcher, 2014; Meyer et al., 2014; Lee et al., 2018). Of these studies, that of Lee et al. (2018) is most relevant to our findings in Loney et al. (2019). Lee et al. (2018) found that the amount of time in which the goal location can be evaluated in the absence of a reward is positively correlated with the development of sign-tracking behavior. As such, delivering the ethanol reward *via* a retractable sipper bottle in Loney et al. (2019) may have prevented the goal location from being evaluated throughout the session, i.e., between the consumption of one reward and the receipt of the next. In turn, we hypothesized that non-static reward delivery (Loney et al., 2019) could bias subjects toward the development of goal-tracking and thereby enhance the effects of nicotine on goal-tracking at the expense of sign-tracking. Additionally, by using a discriminated two-lever design, i.e., including both a predictive and non-predictive lever, in Loney et al. (2019) we thought it possible that nicotine may have had an effect on discrimination learning rather than conditioned approach *per se*. Therefore, in order to address these potential concerns about how the experimental parameters of Loney et al. (2019) may have influenced the development of conditioned approach and biased the effects of nicotine, in experiment 1 of the present study, we assessed the effect of nicotine on conditioned approach triggered by an ethanol-predictive stimulus in a traditional lever-PavCA paradigm. We define a traditional lever-PavCA paradigm as including a single lever and a static reward delivery location, i.e., a fluid receptacle. This experiment was conducted in male Long–Evans rats with and without a history of home-cage ethanol exposure.

Critically, however, Long–Evans rats have been used solely in ethanol-reward PavCA paradigms and not in traditional food-reward PavCA paradigms. As we suggest that nicotine enhances the incentive values of reward cues generally, we hypothesized that the approach responses to both alcohol and food cues would be enhanced. Further, the majority of studies have employed Sprague–Dawley rats; any effect of nicotine found in experiment 1 of the present study could be a result of either rat strain or reward type (natural vs. drug). Therefore, in order to assess whether the same pattern of nicotine’s effect on conditioned approach in response to an ethanol-predictive stimulus could be demonstrated in response to a food-predictive stimulus, in experiment 2, we assessed the effect of nicotine on conditioned approach to a food-predictive stimulus in a lever-PavCA paradigm in male and female Long–Evans rats. Notably, the experimental parameters employed in experiment 2 are identical or strongly similar to those in which an enhancement of sign-tracking has been demonstrated previously in Sprague–Dawley by ourselves (Versaggi et al., 2016) and others (Palmatier et al., 2014; Stringfield et al., 2017), thereby minimizing potential confounds by experimental parameters. Overall, the experiments of the present study sought to reevaluate the effect of nicotine on conditioned approach in response to an ethanol-predictive stimulus under more neutral lever-PavCA experimental parameters and then to investigate whether the pattern of results extended to a food-predictive stimulus, all within Long–Evans rats.

## Materials and Methods

### Subjects and Housing

Long–Evans rats were purchased from Envigo (Indianapolis, IN, United States; experiment 1) or bred at the University at Buffalo (experiment 2). Male rats were used for experiment 1 (*n* = 40; 220–240 g on arrival), whereas male (*n* = 16; 350–450 g) and female (*n* = 16; 220–250 g) rats were used in experiment 2. Rats were housed singly (experiment 1) or doubly (experiment 2) in rectangular cages (45-cm length × 24-cm width × 20-cm height) in a temperature-controlled room (22 ± 1°C) and maintained on a reverse 12-h light–dark cycle (lights on at 9:00 AM for experiment 1 and 10:00 AM for experiment 2; all testing began at least 2 h after the beginning of the dark cycle). Rats were handled daily for 1 week before testing began. Food and water were available *ad libitum* for the duration of all experiments. All procedures were approved by the University at Buffalo Institutional Animal Care and Use Committee.

### Drugs and Injection Procedures

For both experiments, nicotine hydrogen tartrate salt (Glentham Life Sciences, Corsham, United Kingdom) was dissolved in saline, and pH was adjusted to 7.2–7.4 with sodium hydroxide (Matta et al., 2007). Rats were injected subcutaneously with their assigned treatment condition, either nicotine (0.4 mg/kg) or saline, 15 min prior to behavioral testing. Nicotine doses are expressed as the freebase.

### Apparatus

Pavlovian conditioning occurred in Med-Associates chambers (St Albans, VT, United States; 30.5 × 24.1-cm floor area, 29.2 cm high) inside individual sound and light attenuating cabinets (A & B Displays, Bay City, MI, United States) equipped with fans for ventilation and noise-masking. A red house light was located in the center panel of the left wall of the chamber (27 cm above floor). A food and fluid receptacle (2 cm above floor; Med Associates, Model 200R1M-6) was located opposite the house light, in the center panel of the right wall. Entries into the receptacle were detected by interruption of an infrared photo beam. In experiment 1, a syringe pump (Med Associates, PHM-100) mounted outside of the sound attenuating cubicle delivered ethanol (15% vol/vol) in a 10-mL syringe into the receptacle through polyethylene tubing fitted to a stainless-steel cannula (23-gauge). In experiment 2, a pellet dispenser (Med Associates, ENV-203M) delivered banana-flavored food pellets (45 mg, Bio-Serv, Frenchtown, NJ, United States) into the receptacle through feeder tubing (Med Associates, ENV-200-26). In both experiments, a single retractable, illuminated lever (6 cm above floor; Med Associates, Model ENV-112CML) was adjacent to the receptacle, located on either the left or right side. Levers were calibrated such that they were deflected by 15–20 g of pressure. All data were collected using MED-PC IV.2 software.

### Experiment 1: The Effect of Systemic Nicotine on Approach Elicited by an Ethanol-Predictive Stimulus

#### Chronic Intermittent Access to Ethanol

One week after arrival, rats were assigned to either ethanol-exposed or ethanol-naive (water) groups. Exposed rats (*n* = 20) were tested in a chronic intermittent access (CIA) to ethanol paradigm (Wise, 1973; Simms et al., 2008; Carnicella et al., 2014), during which rats were given access to two sipper bottles on their home cages continuously for 24 h. On Monday, Wednesday, and Friday, one bottle contained ethanol (15% vol/vol) and the other tap water, and on the remaining days, both bottles contained tap water. In order to prevent the development of a side preference, ethanol, and water bottles were alternated between the left and right sides of the cages. Naive rats (*n* = 20) were given two bottles containing water on all days. The procedure lasted 28 days, such that ethanol-exposed rats experienced a total of 12, 24-h ethanol drinking sessions. Changes in water and ethanol bottle weights were recorded to determine total ethanol intake (g/kg per 24 h; grams of ethanol consumed per kilogram of body weight over each 24-h session) and ethanol preference (%; ratio of grams of ethanol consumed to total grams of fluid consumed over each 24-h session). To account for fluid loss due to evaporation or spillage, the data for each rat were adjusted by subtracting the average difference in bottle weight from pairs of control ethanol and water bottles placed onto two empty cages. Bottles were weighed and changed daily, with the exception that bottles were not weighed or changed on Sundays. Thus, fluid intake was not recorded for Saturday and Sunday.

#### Chamber Habituation

Rats were habituated to the PavCA testing environment following the 12th session of CIA to ethanol. Habituation occurred on Monday, Wednesday, and Friday. On the first day, rats were brought into the testing room *via* a transfer cart and left in the cart for 20 min before being returned to the holding room. On the second day, rats were transferred to the testing room where they were handled and weighed. Finally, on the third day, rats received a single injection of their assigned drug treatment (see below) in the holding room and were then transferred and placed into the conditioning chambers in the testing room for 20 min. Chambers (including ventilation fans) were turned on during each of these days to habituate the rats to the noise. However, neither the levers nor syringe pumps were turned on during chamber habituation, such that the subjects’ first experience with ethanol in the chamber occurred on the first day of PavCA testing.

#### Treatment Habituation

Rats were assigned to either a nicotine [0.4 mg/kg, subcutaneously (s.c.)] or saline injection treatment group during PavCA and were habituated to their treatment by receiving a single injection of their treatment the day following chamber habituation. These nicotine- and saline-treated groups were matched based on ethanol preference during CIA to ethanol.

#### Ethanol PavCA

**FIGURE 1:**
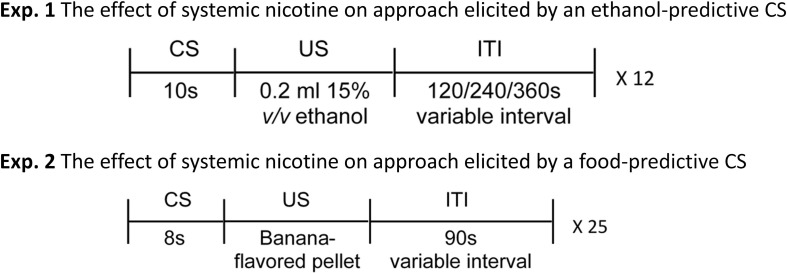
Diagram of the experimental design for experiments 1 and 2. In experiment 1, the 10-s extension of an illuminated lever (CS) predicted the delivery of 0.2 mL ethanol (15% vol/vol; US) into a receptacle. There were 12 CS–US trials, and each trial was separated by an intertrial interval (ITI) of either 120, 240, or 360 s. Rats received an injection of either nicotine (0.4 mL/kg s.c.) or saline 15 min prior to each session. In experiment 2, the 8-s extension of an illuminated lever (CS) predicted the receipt of a single banana-flavored food pellet (US) into a receptacle. There were 25 CS–US trials, and each trial was separated by an ITI on a 90-s (60–150 s) variable time schedule. As in experiment 1, rats in experiment 2 also received an injection of either nicotine (0.4 mL/kg s.c.) or saline 15 min prior to each session.

The first PavCA testing session occurred on the day following treatment habituation. On the morning of each PavCA session, rats were weighed, injected with nicotine or saline, placed into plastic transfer containers on a transfer cart for 15 min, moved to the testing room, and placed into conditioning chambers. Once inside the chamber, rats experienced a 2-min delay, followed by the illumination of the house light, which signaled the start of the session. During the session (Figure 1), the retractable lever (CS) extended into the chamber for 10 s and, upon retraction of the lever, was immediately followed by an 11-s activation of the syringe pump, which dispensed 0.2 mL of ethanol (15% vol/vol; US) into the receptacle. Lever presentations occurred on a variable time (VT) schedule of 140, 260, or 380 s with 12 lever presentations per session. The duration of syringe pump activation was included in the non-CS receptacle entry time period. These parameters were selected in order to closely model those of Srey et al. (2015), with the exception that our syringe pump required 11 s of activation to dispense 0.2 mL of 15% vol/vol ethanol as compared to 6 s. At the end of the session, receptacles were checked for consumption of ethanol, and any remainder was collected *via* syringe and recorded to the nearest 0.2 mL. Sessions occurred on Monday, Wednesday, and Friday, and lasted on average 54 min. Analysis was conducted on the first nine sessions.

### Experiment 2: The Effect of Systemic Nicotine on Approach Elicited by a Food-Predictive Stimulus

#### Exposure and Habituation

Prior to testing in PavCA, rats were given approximately 25 pellets in their home cages on each of two consecutive days. Rats were then assigned to either a nicotine (0.4 mg/kg s.c.) or saline injection treatment group and were habituated to their treatment by receiving a single injection of their treatment on 2 consecutive days. Habituation was separated from pellet exposure by 2 days to avoid conditioned taste aversion. Following habituation, rats underwent receptacle training on two consecutive days, during which they experienced a 5-min blackout period followed by the receipt of 25 banana-flavored food pellets into the receptacle on a VT 30-s (1–60 s) schedule. Fifteen minutes prior to the start of each receptacle training session, all rats received a saline injection, regardless of their assigned injection treatment. Each receptacle training session lasted approximately 17.5 min.

#### Food PavCA

The first PavCA session occurred on the day following receptacle training. On the morning of each PavCA session, rats were weighed, administered their assigned injection treatment, placed into plastic transfer containers (43.5-cm length × 20.3-cm weight × 17.8-cm height) on a transfer cart for 15 min, moved to the testing room, and placed into conditioning chambers. After a 1-min delay, the illumination of the house light signaled the start of the session. During the session (Figure 1), the retractable lever (CS) extended into the chamber for 8 s, and immediately upon retraction of the lever, a single banana-flavored food pellet (US) was delivered into the receptacle. Lever presentations occurred on a VT 90-s (60–150 s) schedule with 25 presentations per session. Receptacles were checked at the end of the session for any uneaten pellets. Sessions were conducted on consecutive days and lasted on average 37.5 min. Analysis was conducted on the first nine sessions.

### Statistical Analysis

#### CIA to Ethanol

A repeated-measures analysis of variance (ANOVA) with *Ethanol Session* (1–12) as the repeated measure was performed on ethanol intake and ethanol preference data.

#### PavCA

For both experiments, in order to assess whether there was a non-specific effect of nicotine on goal location entries, we measured the number of receptacle entries made while the lever was retracted [the intertrial interval (ITI)]. In order to assess nicotine’s effects on the topography of conditioned approach, three aspects of approach to either the lever-CS (sign-tracking) or the receptacle (goal-tracking) were measured: (1) number of lever presses/receptacle entries, (2) probability of a lever-contact/receptacle entry, and (3) latency to contact the lever/enter the receptacle.

For experiment 1, specifically, because there was an effect of nicotine on receptacle entries during the ITI, we separately analyzed the number of receptacle entries that occurred in the 10 s immediately prior to stimulus presentation included as an additional factor (pre-CS entries). As the effect of nicotine was also present in receptacle entries during the pre-CS period, we calculated an elevation score for receptacle entries. This elevation score was calculated by subtracting the number of receptacle entries made during the 10-s pre-CS from the number of receptacle entries made during the 10-s lever presentation. All of these measures from experiment 1 were analyzed in two independent ANOVAs. In the first analysis, a repeated-measures ANOVA was conducted with *Session* (1–9) as the within-groups factor and *Treatment* (nicotine, saline) and *Exposure* (ethanol-exposed, ethanol-naive) in all rats. Thus, for the first analysis, we report the final total subject count for each *Exposure/Treatment* condition (ethanol-exposed/nicotine treated, *n* = 10; ethanol-exposed/saline treated, *n* = 10; ethanol-naive/nicotine treated, *n* = 10; ethanol-naive/saline treated, *n* = 10).

However, a portion of rats (*n* = 9) failed to reliably consume the ethanol reward, i.e., had 1 mL or more of ethanol remaining at the end of session 5. Because none of the significant effects of *Exposure* interacted with *Treatment* in the first analysis and because our primary interest was to evaluate the effect of nicotine in rats that consumed the reward, in a second analysis, a repeated-measures ANOVA was conducted with *Session* (1–9) as the within-groups factor and *Treatment* (nicotine, saline) in all rats that reliably consumed the ethanol reward. While *Exposure* was not included as a factor in the second analysis, we report here the final total subject count for each *Exposure/Treatment* condition (ethanol-exposed/nicotine treated, *n* = 9; ethanol-exposed/saline treated, *n* = 10; ethanol-naive/nicotine treated, *n* = 5; ethanol-naive/saline treated, *n* = 7).

Measures from experiment 2 were analyzed using repeated-measures ANOVA with *Session* (1–9) as the within-groups factor and *Treatment* (nicotine, saline) and *Sex* (male, female) as between-groups factors. Notably, *Sex* never revealed a significant main effect or interaction. For the analysis of experiment 2, the subject count was evenly split with no rats excluded from analysis (nicotine treated/male *n* = 8; saline treated/male, *n* = 8; nicotine treated/female, *n* = 8; saline treated/female, *n* = 8). Significance was determined as *p* < 0.05. *Post hoc* tests were conducted to investigate significant interactions with *Session* and were Bonferroni-corrected such that the significance level was adjusted to correct for multiple comparisons (e.g., if comparing nicotine vs. saline over nine sessions, significance was determined by *p* < 0.05/9 = 0.0056).

## Results

### Experiment 1: The Effect of Systemic Nicotine on Approach Elicited by an Ethanol-Predictive Stimulus

#### Ethanol-Exposed Rats Increased Their Ethanol Intake and Ethanol Preference During CIA

**FIGURE 2:**
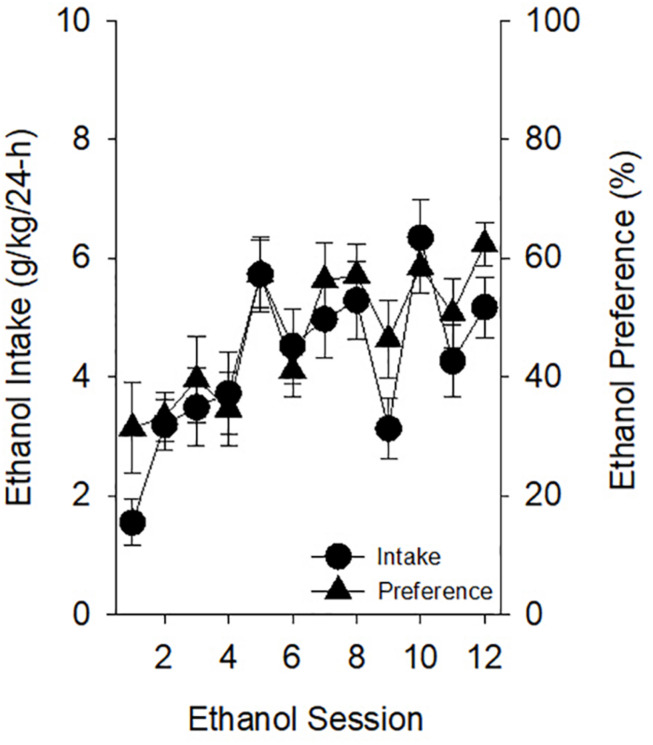
Prior to starting PavCA testing in experiment 1, ethanol-exposed rats increased home-cage ethanol intake and preference. Intakes (grams of ethanol consumed per kilogram of body weight over each 24-h session) and preferences (ratio of grams of ethanol consumed to total grams of fluid consumed over each 24-h session) observed across *Ethanol Sessions* were significantly greater than those on *Ethanol Session 1*. *Post hoc* analyses indicated that ethanol intake was higher on sessions 2–8 and 10–12 than the first ethanol session (*p*’s < 0.0045) and that ethanol preference was significantly higher on sessions 5, 7, 8, and 10–12 relative to the first ethanol session (*p*’s < 0.0045). Data reflect the subjects included in the second ANOVA of experiment 1; thus, one rat of the original 20 ethanol-exposed subjects is excluded.

Ethanol-exposed rats increased their ethanol intake and ethanol preference across CIA sessions [Figure 2; main effects of *Ethanol Session* on ethanol intake (g/kg per 24 h) (*F*_11,198_) = 8.31, *p* < 0.001, and ethanol preference (%) (*F*_11,198_) = 6.93, *p* < 0.001]. *Post hoc* analyses indicated that ethanol intake was higher on sessions 2–8 and 10–12 than the first ethanol session (*p*’s < 0.0045) and that ethanol preference was significantly higher on sessions 5, 7, 8, and 10–12 relative to the first ethanol session (*p*’s < 0.0045).

#### Nicotine Increased Goal Location Entries During the ITI and the 10-s Pre-CS Period

Prior to assessing the effect of nicotine on measures of conditioned approach during presentations of the ethanol-associated stimulus, we sought to determine whether there was evidence for a non-specific effect of nicotine on behavior directed toward the goal location during the ITI. The presence of such a non-specific effect of nicotine would indicate that our measure of goal-tracking should be adjusted to account for increased preoccupation with the goal location during the ITI.

When all rats, regardless of affinity for the ethanol reward, were included in the analysis, nicotine increased the total number of receptacle entries during the ITI relative to saline treatment [main effect of *Treatment* (*F*_1,36_) = 4.76, *p* < 0.05]. This increase varied across sessions [*Treatment* × *Session* interaction (*F*_8,288_) = 2.46, *p* < 0.05]. *Post hoc* analysis revealed differences between nicotine- and saline-treated rats during sessions 4–5 (*p*’s < 0.0056). This effect of nicotine was also present in the number of receptacle entries that were made during the 10-s pre-CS period immediately prior to the presentation of the lever [main effect of *Treatment* (*F*_1,36_) = 5.69, *p* < 0.05].

**FIGURE 3:**
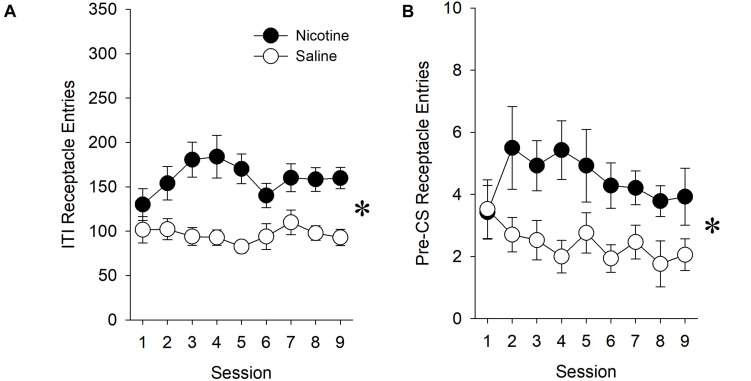
In experiment 1, nicotine increased goal location entries during both the entire ITI and the 10-s pre-CS period immediately prior to lever presentations. In order to determine whether nicotine had a non-specific effect on behavior directed toward the goal location, we assessed the effect of nicotine on receptacle entries during both the entire ITI **(A)** and during the 10-s pre-CS period **(B)**. Because nicotine increased receptacle entries during both the ITI and the pre-CS period in experiment 1, we express the number of receptacle entries in our evaluation of goal-tracking (Figure 4A) as an elevation score, as opposed to raw values. The elevation score was calculated by subtracting the number of receptacle entries made during the 10-s pre-CS from the number of receptacle entries made during the 10-s lever presentation. Asterisk (^∗^) indicates significant *Treatment* × *Session* interactions.

Similarly, when only rats with an affinity for the ethanol reward were included in the analysis, nicotine increased ITI receptacle entries relative to saline treatment [Figure 3A; main effect of *Treatment* (*F*_1,29_) = 21.87, *p* < 0.001]. This effect of nicotine was also present in the number of receptacle entries that were made during the 10-s pre-CS period immediately prior to the presentation of the lever (Figure 3B; main effect of *Treatment* (*F*_1,29_) = 16.03, *p* < 0.001. However, there was no *Treatment* × *Session* interaction.

Therefore, in order to account for this non-specific effect of nicotine on behavior directed toward the goal location during the ITI, we express the number of receptacle entries in our evaluation of goal-tracking as an elevation score, as opposed to as raw values.

#### Nicotine Strengthened Goal-Tracking in Response to an Ethanol-Predictive Stimulus, Even After Accounting for the Effect of Nicotine on ITI Goal Entries

##### Number of Receptacle Entries

In order to account for the increased activity demonstrated by nicotine-treated animals during the ITI, we computed an elevation score, in which the number of receptacle entries made dung the 10-s pre-CS period was subtracted from the number of receptacle entries made during the 10-s CS presentation.

When all rats, regardless of affinity for the ethanol reward, were included in the analysis, nicotine treatment was found to vary across sessions [*Treatment* × *Session* interaction (*F*_8,288_) = 2.50, *p* < 0.05], with *post hoc* analysis revealing that nicotine increased the number of goal-tracking responses relative to saline treatment during session 6 (*p* < 0.0056). Notably, ethanol exposure also continued to independently increase the number of receptacle entries relative to ethanol naivety [main effects of *Exposure* (*F*_1,36_) = 7.84, *p* < 0.05; and *Session* (*F*_8,288_) = 27.29, *p* < 0.001]. The effect of exposure was found to vary across sessions [*Exposure* × *Session* interaction (*F*_8,288_) = 2.24, *p* < 0.05]. *Post hoc* analysis revealed differences between ethanol-exposed and ethanol-naive rats during sessions 5, 6, 8, and 9 (*p*’s < 0.0056).

**FIGURE 4:**
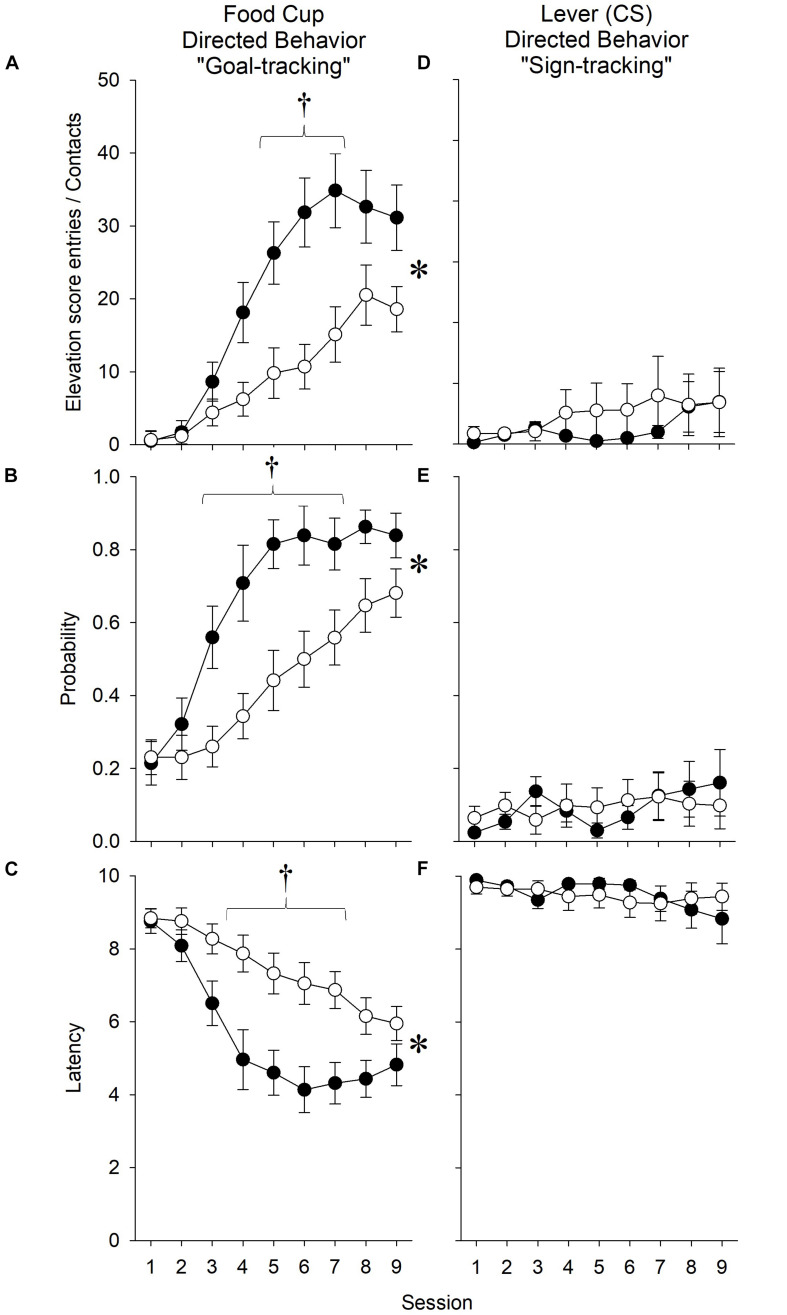
In experiment 1, nicotine enhanced measures of goal-tracking elicited by an ethanol-predictive stimulus relative to saline treatment. During the extension of a lever that predicted the non-contingent receipt of ethanol (15% vol/vol), nicotine **(A)** increased the number of receptacle entries as calculated by the elevation score, **(B)** increased the probability of making a receptacle entry, and **(C)** decreased the latency to the first receptacle entry. Conversely, nicotine did not affect **(D)** the number of lever presses, **(E)** the probability of contacting the lever, or **(F)** the latency to the first lever contact. Asterisk (^∗^) indicates significant *Treatment* × *Session* interactions, whereas daggers (^†^) indicate significant differences on the indicated test sessions. Data reflect the subjects included in the second ANOVA of experiment 1; thus, rats that did not have an affinity for ethanol are excluded and are collapsed across *Ethanol Exposure*.

When only rats with an affinity for the ethanol reward were included in the analysis, nicotine increased the number of receptacle entries relative to saline treatment [Figure 4A; main effects of *Treatment* (*F*_1,29_) = 10.13, *p* < 0.05; and *Session* (*F*_8,232_) = 34.44, *p* < 0.001]. The effect of nicotine treatment was found to vary across sessions [*Treatment* × *Session* interaction (*F*_8,232_) = 4.99, *p* < 0.001]. *Post hoc* analysis revealed differences between nicotine- and saline-treated rats during sessions 5–7 (*p*’s < 0.0056).

Therefore, even when accounting for a non-specific effect of nicotine on preoccupation with the goal location during the ITI, nicotine enhanced the number of goal-tracking responses performed in response to the ethanol-associated stimulus.

##### Probability of Receptacle Entry

As a second assessment of nicotine’s effect on goal-tracking, we evaluated the effect of nicotine on the probability of rats to make a goal-tracking response on any given trial.

When all rats, regardless of affinity for the ethanol reward, were included in the analysis, ethanol exposure, relative to ethanol naivety, increased the probability of receptacle entry [main effects of *Exposure* (*F*_1,36_) = 9.34, *p* < 0.05; and *Session* (*F*_8,288_) = 27.61, *p* < 0.001]. Nicotine increased the probability of receptacle entry across sessions relative to saline treatment [*Treatment* × *Session* interaction (*F*_8,288_) = 2.65, *p* < 0.05], although *post hoc* analysis did not reveal differences during specific sessions.

When only rats with an affinity for the ethanol reward were included in the analysis, nicotine increased the probability of receptacle entries relative to saline treatment [Figure 4B; main effects of *Treatment* (*F*_1,29_) = 9.36, *p* < 0.05; and *Session* (*F*_8,232_) = 34.00, *p* < 0.001]. The effect of nicotine treatment was found to vary across sessions [*Treatment* × *Session* interaction (*F*_8,232_) = 3.67, *p* < 0.001. *Post hoc* analysis revealed differences between nicotine- and saline-treated rats during sessions 3–6 (*p*’s < 0.0056).

Therefore, nicotine enhanced the proportion of trials in which a goal-tracking response occurred.

##### Latency to Receptacle Entry

As a third assessment of nicotine’s effect on goal-tracking, we evaluated the effect of nicotine on the latency to make a goal-tracking response upon presentation of the ethanol-associated stimulus.

When all rats, regardless of affinity for the ethanol reward, were included in the analysis, ethanol exposure relative to ethanol naivety decreased the latency of receptacle entry [main effect of *Exposure* (*F*_1,36_) = 11.53, *p* < 0.05]. Nicotine decreased the latency of receptacle entry across sessions relative to saline treatment [*Treatment* × *Session* interaction (*F*_8,288_) = 2.70, *p* < 0.05], although *post hoc* analysis did not reveal differences during specific sessions. The effect of ethanol exposure was also found to vary across sessions [*Exposure* × *Session* interaction (*F*_8,288_) = 2.05, *p* < 0.05]. *Post hoc* analysis revealed differences between ethanol-exposed and ethanol-naive rats during sessions 4–6 (*p*’s < 0.0056).

When only rats with an affinity for the ethanol reward were included in the analysis, nicotine decreased the latency of receptacle entries relative to saline treatment [Figure 4C; main effects of *Treatment* (*F*_1,29_) = 10.93, *p* < 0.05; and *Session* (*F*_8,232_) = 26.84, *p* < 0.001]. The effect of nicotine treatment was found to vary across sessions [*Treatment* × *Session* interaction (*F*_8,232_) = 3.99, *p* < 0.001. *Post hoc* analysis revealed differences between nicotine- and saline-treated rats during sessions 4–7 (*p*’s < 0.0056).

Therefore, nicotine decreased the latency to make the first goal-tracking response.

#### Nicotine Had No Effect on Sign-Tracking in Response to an Ethanol-Predictive Stimulus

In order to assess the effect of nicotine on sign-tracking, we conducted both of the aforementioned ANOVAs on the number of sign-tracking responses during the presentation of the ethanol-associated stimulus, the probability to make a sign-tracking response, and latency to the first sign-tracking response. There were no significant effects of nicotine treatment or ethanol exposure on any measure (Figures 4D–F).

### Experiment 2: The Effect of Systemic Nicotine on Approach Elicited by a Food-Predictive Stimulus

#### Nicotine Had No Effect on the Number of Receptacle Entries During the ITI

**FIGURE 5:**
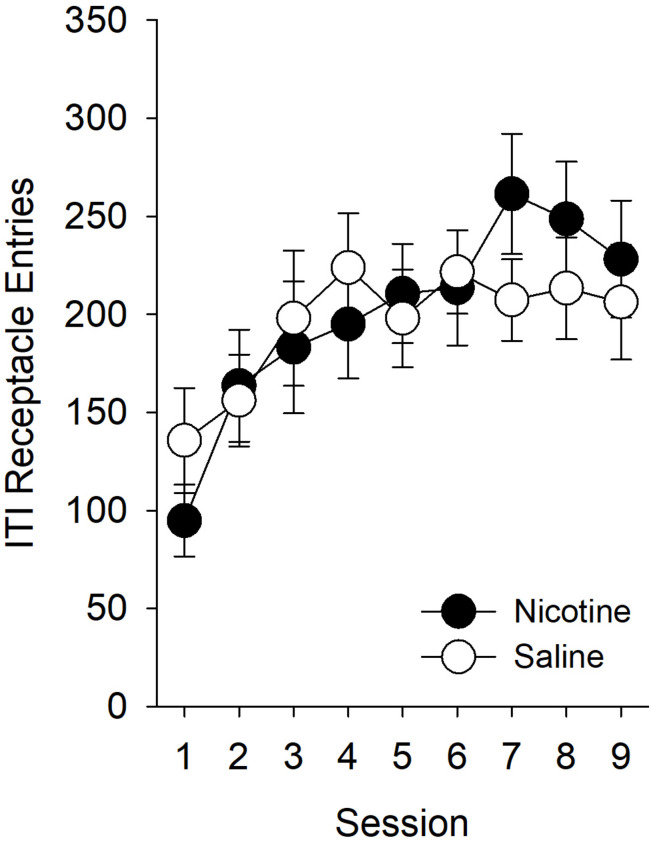
In experiment 2, nicotine had no effect on the number of goal location entries during the ITI. As in experiment 1, we sought to determine whether nicotine had any non-specific effect on behavior directed toward the goal location by evaluating the effect of nicotine on receptacle entries during the ITI. In this experiment, nicotine did not have any effect on the number of receptacle entries during the ITI. Therefore, we did not compute an elevation score for experiment 2 and report the raw values for the number of goal-tracking responses in Figure 6A.

As in experiment 1, we first assessed whether there was a non-specific effect of nicotine on behavior directed toward the goal location during the ITI. While ITI entries increased across sessions [Figure 5; main effect of *Session* (*F*_8,224_) = 6.03, *p* < 0.001], there were no effects of, or interactions with, *Treatment* or *Sex*. Therefore, we did not compute an elevation score for experiment 2.

#### Nicotine Increased Goal-Tracking in Response to a Food-Predictive Stimulus

##### Number of Receptacle Entries

**FIGURE 6:**
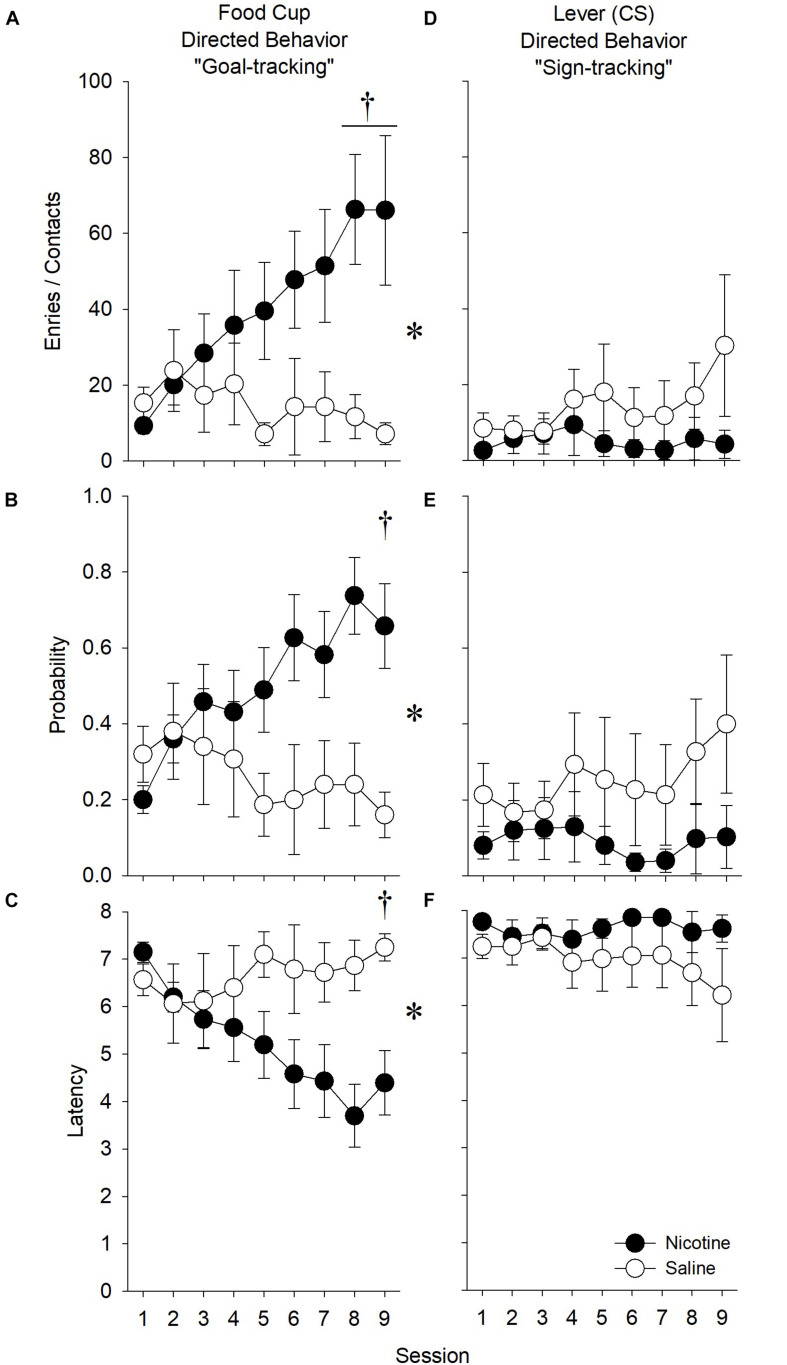
In experiment 2, nicotine enhanced measures of goal-tracking elicited by a food-predictive stimulus relative to saline treatment. During the extension of a lever that predicted the non-contingent receipt of a solid food reward, nicotine **(A)** increased the number of receptacle entries, **(B)** increased the probability of making a receptacle entry, and **(C)** decreased the latency to the first receptacle entry. Conversely, nicotine **(D)** did not affect the number of lever presses, **(E)** tended to reduce the probability of contacting the lever, and **(F)** did not affect the latency to the first lever contact. The effect of nicotine did not differ by *Sex* for any measure. Asterisk (^∗^) indicates significant *Treatment* × *Session* interactions, whereas daggers (^†^) indicate significant differences on the indicated test sessions.

In order to assess the effect of nicotine on goal-tracking, we analyzed the effect of nicotine on the raw number of goal-tracking response made during the presentation of the food-predictive stimulus. Nicotine increased the number of receptacle entries relative to saline treatment across sessions [Figure 6A; main effects of *Treatment* (*F*_1,28_) = 4.44, *p* < 0.05; and *Session* (*F*_8,224_) = 32.89, *p* < 0.001; *Treatment* × *Session* interaction (*F*_8,224_) = 5.30, *p* < 0.001]. *Post hoc* analysis indicated that this enhancement occurred during sessions 8 and 9 (*p*’s < 0.0056). Further, males goal-tracked more than females across sessions [main effect of *Sex* (*F*_1,28_) = 6.86, *p* = 0.01; *Sex* × *Session* interaction (*F*_8,224_) = 2.41, *p* < 0.05]. *Post hoc* analysis revealed that males goal-tracked more than females during sessions 8 and 9 (*p*’s < 0.0056). However, there were no significant interactions between *Sex* and *Treatment*. Thus, nicotine increased the amount of goal-tracking responses similarly in male and female rats.

##### Probability of Receptacle Entry

As in experiment 1, we also assessed the effect of nicotine on the probability to make a goal-tracking response. Nicotine increased the probability of receptacle entry across sessions relative to saline treatment [Figure 6B; main effect of *Session* (*F*_8,224_) = 32.18, *p* < 0.01; *Treatment* × *Session* interaction (*F*_8,224_) = 3.38, *p* < 0.01]. *Post hoc* analysis revealed significant differences at session 9 (*p*’s < 0.0056). There was no main effect of or interactions with *Sex*.

##### Latency to Receptacle Entry

As in experiment 1, we assessed the effect of nicotine on the latency to the first goal-tracking response. Nicotine decreased the latency of receptacle entry across sessions relative to saline treatment [Figure 6C; main effect of *Session* (*F*_8,2__24_) = 30.86, *p* < 0.01; *Treatment* × *Session* interaction (*F*_8,224_) = 2.19, *p* < 0.05]. *Post hoc* analysis revealed differences during session 9 (*p*’s < 0.0056). There was no main effect of or interaction with *Sex*.

#### Nicotine Had No Effect on Sign-Tracking in Response to a Food-Predictive Stimulus

In order to assess the effect of nicotine on sign-tracking, we analyzed the number of sign-tracking responses during the presentation of the ethanol-associated stimulus, the probability to make a sign-tracking response, and latency to the first sign-tracking response. There were no significant effects of *Treatment* or *Sex* on any measure (Figures 6D–F).

## Discussion

### Summary

Here, we report that nicotine enhances measures of goal-tracking, without affecting measures of sign-tracking, in lever-PavCA paradigms wherein the reward delivery location remains static. Specifically, in experiment 1, when a lever predicted an ethanol reward, nicotine increased the number of goal-tracking responses and decreased the latency to the first goal-tracking response in male rats with and without a history of home-cage ethanol exposure. Further, in experiment 2, when a lever predicted a food reward, nicotine increased the number and probability of goal-tracking responses, decreased the latency to the first goal-tracking response, and was associated with stronger goal-tracking phenotypes in both male and female rats. These findings expand upon our earlier work by demonstrating that the effect of nicotine on goal-tracking observed in Loney et al. (2019) was not due to the non-static nature of the location of the ethanol reward biasing the development of goal-tracking, which was an important consideration, given that the amount of time in which the goal location can be evaluated in the absence of the reward is positively correlated with the development of sign-tracking (Lee et al., 2018). Thus, nicotine’s effects on conditioned approach are more robust than previously supported and are inconsistent with the hypothesis that nicotine facilitates approach to reward-predictive stimuli. In turn, current theory of the mechanisms by which nicotine affects conditioned approach must be expanded.

### Nicotine’s Effects on Conditioned Approach Triggered by Ethanol-Predictive Stimuli

We have recently shown that nicotine enhances goal-tracking triggered by an ethanol-predictive stimulus (Loney et al., 2019) when the reward delivery location was non-static. However, several experimental parameters, including the nature of the stimulus (Hearst and Jenkins, 1974; Meyer et al., 2014) and of the reward (Hearst and Jenkins, 1974; Uslaner et al., 2006), the duration of the stimulus, reward, and ITI (Hearst and Jenkins, 1974; Lee et al., 2018), the relative locations of the stimulus and of the reward (Palmatier et al., 2013), the number of testing sessions (Srey et al., 2015; Villaruel and Chaudhri, 2016), and the timing of the introduction of nicotine during testing (Guy and Fletcher, 2014), are well-known to influence the topography of conditioned responding in PavCA paradigms. These parameters can vary considerably between laboratories, impeding replication in a paradigm that attempts to maximize the identification of individual differences. Further, the extent to which such parameters interact with inherent individual differences is largely unknown and likely variable. Because of these considerations in general and the findings of Lee et al. (2018) specifically, we felt it necessary to reevaluate our work in Loney et al. (2019). Namely, given that the evaluation of the goal location in the absence of the reward is related to the development of sign-tracking (Lee et al., 2018), we felt that we needed to determine whether the non-static nature of the ethanol delivery location in our previous study could have engendered the development of goal-tracking behavior and thus biased an effect of nicotine on goal-tracking. We found that nicotine also enhances goal-tracking triggered by an ethanol-predictive stimulus in a lever-PavCA paradigm where the reward delivery location was static. Nonetheless, we could not address all of the known factors that could influence conditioned approach and thus potentially nicotine’s effect. Namely, there is some evidence to suggest that extending the number of testing sessions could “shift” the conditioned response from goal- to sign-tracking (Srey et al., 2015, but also see Villaruel and Chaudhri, 2016). Further, there is substantial literature demonstrating differences in the effect of experimenter- vs. self-administered nicotine, as well as distinct differences in ethanol intake as a function of relation to the time of nicotine administration (Le et al., 2000, 2003, 2014; Olausson et al., 2001; Sharpe and Samson, 2002; Metaxas et al., 2010; Bito-Onon et al., 2011; Donny et al., 2011; Hauser et al., 2012; Doyon et al., 2013). These parameters are worthy of further investigation in future studies. We note, however, that, while our experimental parameters are not identical to any current existing study, they do reasonably follow those of at least one other ethanol PavCA study (Srey et al., 2015; Maddux and Chaudhri, 2017) and in this way aim to contribute to a consistent approach within the field. Overall, indicate that nicotine can facilitate behaviors elicited by ethanol-associated stimuli without increasing approach to those stimuli.

It is surprising that we did not find an effect of nicotine on sign-tracking triggered by an ethanol-predictive stimulus, considering that multiple studies have shown that an ethanol reward can engender sign-tracking (Krank et al., 2008; Srey et al., 2015; Villaruel and Chaudhri, 2016). Thus, it is possible that the interaction between nicotine and ethanol alters the properties by which ethanol alone produces sign-tracking. For example, we have found that nicotine can reduce the aversive postingestive consequences of intoxication (Loney and Meyer, 2019; Loney et al., 2019) and increase the acceptance of increasing ethanol concentrations (Loney and Meyer, 2018). Such effects could change the relative reinforcing value of the ethanol reward during PavCA and consequently alter the expression of the conditioned response. However, that we also did not observe sign-tracking in our saline-treated rats suggests that the effect of nicotine on conditioned approach may reflect certain qualities of our cohort rather than unique interactions between nicotine and ethanol. Indeed, there is strong support for a role of genetic composition and heritability in the expression of PavCA phenotypes (Fitzpatrick et al., 2013; Koshy Cherian et al., 2017). Because only 2 of the 34 rats included in experiment 1 expressed a sign-tracking phenotype, the majority of our cohort may have been predisposed to express goal-tracking phenotypes. Thus, one possibility as to why we observed an effect of nicotine on goal-tracking is that genetic composition affects whether nicotine will enhance goal- or sign-tracking phenotypes, an idea also expressed by Stringfield et al. (2017). This could also explain why we were unable to detect an effect of nicotine in Index Score in experiment 1, in that there was a ceiling effect on the extent to which nicotine could increase an already biased goal-tracking disposition. It is also possible that, whereas there is some evidence that under certain conditions Long–Evans rats do engage in sign-tracking (Srey et al., 2015), Long–Evans rats may be more predisposed to goal- than sign-track, especially with respect to Sprague–Dawley rats where the proportions of intermediates and sign- and goal-trackers have been established as being quite evenly distributed (Meyer et al., 2012). If true, this could be concerning in that ethanol PavCA studies to date have only been conducted in Long–Evans rats (Srey et al., 2015; Maddux and Chaudhri, 2017; Loney et al., 2019), and therefore, it is likely necessary to conduct ethanol, as well as nicotine and ethanol, PavCA studies using Sprague–Dawley rats as well. Here, our assertion that nicotine affects goal-tracking conditioned approach is strengthened by our demonstration of this effect in Long–Evans rats for both ethanol and food reinforcers, thereby reducing the possibility that nicotine’s effect is resultant of the combination of strain and type of reinforcer.

### Nicotine’s Effects on Conditioned Approach Triggered by Food-Predictive Stimuli

Although we and others have previously shown that nicotine enhances sign-tracking triggered by food- and water-predictive stimuli in male rats (Palmatier et al., 2014; Versaggi et al., 2016; Stringfield et al., 2017), the present findings demonstrate that nicotine can enhance goal-tracking in male and female rats under identical experimental parameters. Taken together, we can conclude that it is sufficient, but not necessary, that nicotine amplifies the incentive motivational properties of food-predictive stimuli in order to facilitate approach behavior. These diverse effects lend support to the idea that genetic composition and heritability may determine the effect of nicotine on conditioned approach. However, in experiment 2, we found significant between-groups differences on Index Score. Review of the subjects within each group suggests that there was more individual variability in saline- than nicotine-treated rats. This could either be a result of random group assignment or suggest that nicotine may produce a bias for stronger goal-tracking phenotypes, at least under some experimental parameters. Such a bias has been observed after amphetamine administration (Holden and Peoples, 2010; DiFeliceantonio and Berridge, 2016). Specifically, amphetamine administration into the dorsolateral striatum increased goal-tracking in rats that displayed goal-tracking or non-exclusive sign-tracking at baseline, but did not increase goal-tracking in rats that exclusively displayed sign-tracking at baseline (DiFeliceantonio and Berridge, 2016). Unfortunately, the design of our current study did not include a baseline assessment of each individual’s predisposed phenotype, and it has been suggested that the effect of nicotine differs as a function of administration early or late in training (Guy and Fletcher, 2014). However, no effect of nicotine on sign-tracking triggered by any reward-predictive stimulus has been demonstrated in Long–Evans rats, therefore leaving open the possibility of a strain specific bias for nicotine, primarily altering goal-tracking.

### Does Nicotine Enhance Goal-Tracking by Incentive Motivational Mechanisms?

Sign-tracking is considered to be a result of the attribution of incentive motivational value to reward stimuli (Flagel et al., 2011), in which the incentive value of these stimuli affects behavior independently of the incentive value of the reward itself. This leads to inflexible responding and insensitivity to omission conditions (Lovic et al., 2011; King et al., 2016; Meyer and Tripi, 2018). The psychological mechanism underlying goal-tracking, however, has been subject to a more open-ended debate. While goal-tracking behavior has been shown to be flexible and able to be withheld under reward omission and US devaluation conditions (Ahrens et al., 2016; Meyer and Tripi, 2018; Keefer et al., 2020), nicotine has been shown to reduce such sensitivity to US manipulations (Stringfield et al., 2018; Loney and Meyer, 2019; Loney et al., 2019). Thus, in contrast to the conceptualization of goal-tracking as a flexible behavioral response, the enhancing effect of nicotine in the current studies suggests that goal-tracking is influenced by other psychological processes as well.

For example, goal-tracking may reflect goal-directed behavior based on cognitive expectations of the availability of a reward. Support for this hypothesis has come from Sarter and colleges, who have shown extensive differences between sign- and goal-trackers in prefrontal dopaminergic and cholinergic modulator systems (Koshy Cherian et al., 2017; Pitchers et al., 2017a). Goal-trackers have been characterized by superior cholinergic functioning (Koshy Cherian et al., 2017; Pitchers et al., 2017b) and consequently superior performance in tasks of attention (Paolone et al., 2013). Notably, this system can be artificially modulated by nicotine, albeit less efficiently than strict α4β2 agonists (Howe et al., 2010). As such, nicotine could also strengthen goal-tracking by augmenting an aspect of learning *via* modulation of attention, leading to increased anticipation of reward receipt or expectation of reward availability.

At the same time, Berridge and colleagues have suggested that both sign-tracking and goal-tracking reflect increases in incentive motivation. They demonstrated that under conditions of mesolimbic activation, such as by dopamine or synthetic mu opioid activation, goal-tracking can become “frenzied” such as to also reflect incentive motivational mechanisms (Mahler and Berridge, 2009; DiFeliceantonio and Berridge, 2012, 2016). Under this situation, nicotine administration would cause a long-lasting state of mesolimbic activation that would essentially set the stage for encounters with reward-associative stimuli to acutely narrow motivational focus onto a conditioned stimulus. Notably, the trigger for this phasic burst of incentive motivation would always result from exposure to the reward-predictive stimulus. However, the target of that incentive motivation could vary between individuals, such that their predisposition to sign- or goal-track would guide whether their “prepotent” conditioned stimulus associated with the receipt of the reward. Thus, in sign-trackers, the target would be the reward-predictive stimulus itself, whereas in goal-trackers the target would be the reward-contiguous stimulus, i.e., the location of reward delivery. Further, they argue that, through this process, habits could also acquire a “must-do” emphasis transforming their habitual nature to a motivated process (DiFeliceantonio and Berridge, 2016). This is perhaps the most parsimonious explanation for the whole of nicotine’s effects on conditioned approach. Indeed, this explanation accounts for the variability of nicotine’s effects on both sign- and goal-tracking processes, although the underlying underpinnings of the individual differences that contribute to an individual’s “prepotent” conditioned stimulus are largely yet to be determined. Possible sources of these individual differences, as mentioned, likely involve heritable factors generally (Fitzpatrick et al., 2013) and cholinergic factors specifically (Sarter et al., 2016a; Koshy Cherian et al., 2017). In addition, the description of time-locked, phasic increases in incentive motivation coincides with the ITI data in the present study. Specifically, increases in interaction with the goal were observed during lever presentations and not during the pre-CS period in experiment 1 or during the ITI in experiment 2, even though the location of the reward was available for evaluation for the duration of the session. In summary, the mechanisms by which nicotine facilities behavior may be best understood as an individually determined effect of nicotine on the incentive-amplifying properties of either reward-predictive or reward-contiguous stimuli.

### Conclusion

Although there is substantial evidence that nicotine indeed can facilitate behavior by acting as an incentive amplifier of reward-predictive stimuli, our findings demonstrate that additional mechanisms by which nicotine may affect behavior must be considered. More work is required to determine which specific psychological mechanisms, as well as their neurobiological underpinnings, are affected on an individual basis. Specifically, future work will address (1) whether these effects of nicotine are mediated by nicotinic receptors within the brain, (2) whether cholinergic circuits within the forebrain promote conditioned approach in the absence of nicotine, and (3) whether nicotine promotes conditioned approach through cortical or subcortical brain systems, or both.

## Data Availability Statement

The raw data supporting the conclusions of this article will be made available by the authors, without undue reservation.

## Ethics Statement

The animal study was reviewed and approved by The University at Buffalo Institutional Animal Care and Use Committee.

## Author Contributions

HA, GL, and PM contributed conception and design of the study and edited the manuscript. HA and GL conducted the experiments. HA organized the database, performed the statistical analysis, and wrote the first draft of the manuscript. All authors contributed to manuscript revision, read, and approved the submitted version.

## Conflict of Interest

The authors declare that the research was conducted in the absence of any commercial or financial relationships that could be construed as a potential conflict of interest.

## Publisher’s Note

All claims expressed in this article are solely those of the authors and do not necessarily represent those of their affiliated organizations, or those of the publisher, the editors and the reviewers. Any product that may be evaluated in this article, or claim that may be made by its manufacturer, is not guaranteed or endorsed by the publisher.
